# Effect of Postoperative Oral Intake on Prognosis for Esophageal Cancer

**DOI:** 10.3390/nu11061338

**Published:** 2019-06-14

**Authors:** Genya Okada, Chika Momoki, Daiki Habu, Chisako Kambara, Tamotsu Fujii, Yasunori Matsuda, Shigeru Lee, Harushi Osugi

**Affiliations:** 1 Department of Health Sciences, Prefectural University of Hiroshima, 1-1-71 Ujina-higashi, Minami-ku, Hiroshima 734-8558, Japan; kambara@pu-hiroshima.ac.jp (C.K.); fujii@pu-hiroshima.ac.jp (T.F.); 2 Department of Nutritional Medicine, Graduate School of Human Life Science, Osaka City University, 3-3-138 Sugimoto-cho, Sumiyoshi-ku, Osaka 558-8585, Japan; momoki@tezukayama-u.ac.jp (C.M.); habu@life.osaka-cu.ac.jp (D.H.); 3 Department of Food and Nutrition, Faculty of Contemporary Human Life Science, Tezukayama University, 3-1-3 Gakuenminami, Nara 631-8585, Japan; 4 Department of Gastroenterological Surgery, Graduate School of Medicine, Osaka City University, 1-4-3 Asahi-machi, Abeno-ku, Osaka 545-8585, Japan; m1293311@msic.med.osaka-cu.ac.jp (Y.M.); m3010375@msic.med.osaka-cu.ac.jp (S.L.); osugi.harushi@twmu.ac.jp (H.O.); 5 Institute of Gastroenterology, Tokyo Women’s Medical University, 8-1 Kawada-cho, Sinjuku-ku, Tokyo 162-8666, Japan

**Keywords:** esophagectomy, energy intake, nutrition therapy, administration, outcomes research/quality

## Abstract

Background: Patients undergoing surgery for esophageal cancer are at risk of prolonged hospital stay for postoperative malnutrition. Postoperative early oral feeing is a part of the “enhanced recovery after surgery protocol” for coping with this risk. However, the usefulness of early oral intake during perioperatively is questionable. Methods: In total, 117 patients treated surgically for esophageal cancer were analyzed in the study. We assessed the oral energy sufficiency rate per nutritional requirement (oral-E/NR) at the fourth week postoperatively and classified the patients into two groups: Poor oral intake group (POI group; <25% oral-E/NR) and the control group (≥25% oral-E/NR). We analyzed the relationship among postoperative oral intake and prognoses. Results: The POI group had worse postoperative nutritional status and a lower survival rate than the control group. In a multivariate analysis, <25% oral-E/NR was one of the independent factors contributing to negative outcomes postoperatively (adjusted hazard ratio: 2.70, 95% confidence interval: 1.30–5.61). Conclusions: In patients undergoing surgery for esophageal cancer, poor postoperative oral intake negatively affected not only on their postoperative nutritional status but also their overall prognosis. It is necessary to improve the adequacy of oral intake postoperatively for patients with esophageal cancer.

## 1. Introduction

Esophagectomy is one of the most invasive surgeries performed in patients with esophageal cancer; in addition to tumor resection, this surgery requires a wide operative field with lymph node dissection and resection. Because the function of the esophagus affects the metabolic, neuroendocrine, and immune systems, the nutritional status significantly deteriorates postoperatively because of hypermetabolism and digestion-absorption disorders [1,2,3,4,5,6,7,8]. In addition, oral intake is considerably diminished after the operation because of the mechanical and functional gastrointestinal tract changes that cause swallowing disorders, early satiety, and postprandial dumping syndrome [9].

Therefore, some patients treated surgically for esophageal cancer require supplemental nutritional management using enteral and parenteral nutrition to fulfill their nutritional requirements (NRs) during perioperatively. On the other hand, in order to shorten postoperative hospital stays, patients often get discharged after fulfilling NRs using enteral nutrition (EN) and parenteral nutrition (PN) only, despite still displaying inappropriate oral intake.

The most general algorithms for nutrition administration have recommended that oral intake, the physiological food administration route, be re-established as soon as possible and that it should take priority over EN and PN [10,11]. Based on these guidelines, studies on nutrition administration in critically ill patients have questioned the nutrition quantity and timing of administration using EN and PN [12,13,14,15]. Furthermore, several studies have addressed the topic of efficacious postoperative direct oral feeding as part of the “enhanced recovery after surgery (ERAS) protocol [16]”. Several studies have reported that patients undergoing surgery for esophageal cancer resumed oral feeding at postoperative meal start [postoperative day (POD) 1] [17,18], and the importance of oral intake is increasing. In the present study, we enrolled patients between June 2009 and December 2010, and some differences existed due to the current trends of nutritional support. As we had been in the process of shifting the major nutritional management from total parenteral nutrition (TPN) to gut (EN and oral), we secured TPN and EN routes (standardizing at the time) in almost all patients at our hospital, and nutrition management was primarily based on EN.

The cephalic phase responses (CPRs) can explain the benefits of oral intake because they prepare the gastrointestinal tract for digestion and absorption [19,20,21]. However, no studies have assessed whether postoperative nutritional management including oral intake affects short-term and long-term outcomes in patients with esophageal cancer.

In this study, we re-analyzed the patients enrolled in our previous prospective study [22] to investigate the relationship between postoperative oral intake and prognosis, because we obtained substantial follow-up data in these patients.

## 2. Participants and Methods

### 2.1. Participants

This was a retrospective study re-analyzing patients enrolled in our previous prospective study [22] to investigate the correlation between postoperative oral intake and prognosis, after obtaining substantial follow-up data (five-year survival) of these patients. All patients were diagnosed with esophageal cancer and signed informed consents before participating in the study that was approved by the Ethics Committee of Osaka City University Medical School who approved the study (No.1611). All procedures followed were in accordance with the ethical standards of the responsible committee on human experimentation (institutional and national) and with the Helsinki Declaration of 1964 and later versions.

In the present study, the inclusion criteria were as follows: (1) Patients with thoracic esophageal cancer undergoing elective surgical treatment (irrespective of the tumor stage, operation procedure, or esophageal reconstruction) between June 2009 and December 2010. Conversely, the exclusion criteria were as follows: (1) Emergency esophagectomy; (2) cervical esophageal cancer requiring pharyngolaryngectomy; (3) severe cardiac, liver, or renal failure; or (4) patients in whom the nutrient intake quantity could not be recorded.

All patients underwent standard open esophagectomy through a conventional right posterolateral incision in the bed of the resected the fifth rib or video-assisted thoracoscopic esophagectomy (VATS) in Osaka City University Hospital. Details of the surgical techniques have been published elsewhere [22,23]. Reconstruction was performed using a gastric conduit or a jejunal graft through the posterior mediastinum. A jejunostomy was inserted for postoperative enteral feeding.

### 2.2. Perioperative Nutritional Management

We explained the importance of nutritional management to the patients before their hospital admission to ensure their adequate nutritional status using oral, enteral, and parenteral routes during the preoperative period. Once admitted to the hospital, we recorded the quantity of nutrient intake (energy and protein) by measuring the daily oral intake (only hospital diet), EN, and PN during the perioperative period. The daily oral intake was assessed by calorie count (% of plate eaten for oral diet). These data were used to calculate the average nutrient intake for every week. During the postoperative period, we calculated the NRs using the following formula: NR (kcal/day) = Basal Energy Expenditure (BEE) × 1.3. BEE was calculated using the Harris–Benedict equation (Table S1). We used oral, enteral, and parenteral routes to try to satisfy the resulting NRs. In addition, we have collated the events associated meal interruption during postoperative hospital stay.

### 2.3. Classification According to Postoperative Oral Intake

**Figure 1:**
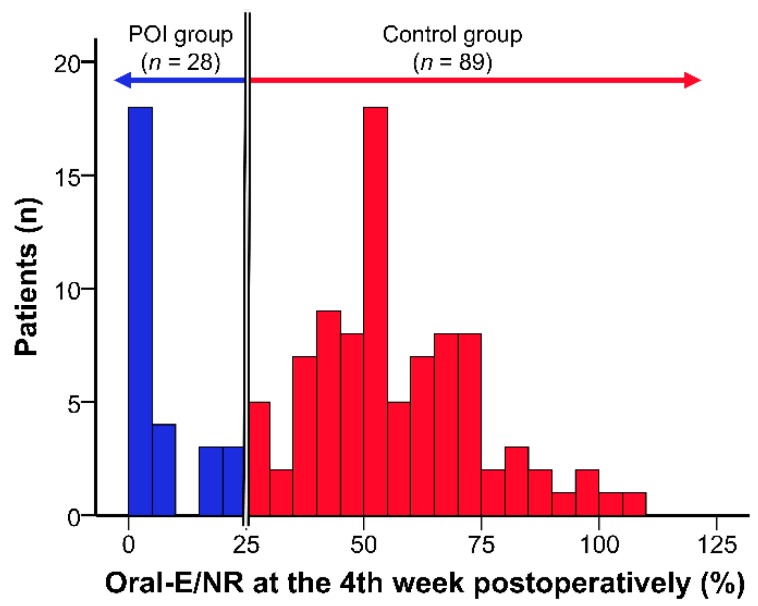
The distribution of oral-E/NR at the 4th week postoperatively.

In order to assess the quality of nutrition during the median hospital stay of 28 days, we calculated the mean amount and adequacy of energy administered per NR for each administration route at the fourth week postoperatively [for early discharge cases, we used data obtained at the third week postoperatively (*n* = 31)]. The distribution of values of oral energy intake per NR (oral-E/NR) at the fourth week postoperatively is shown in Figure 1. We classified the patients according to the 25th percentile value of oral-E/NR (26.6%); patients with oral-E/NR ≥ 25% were assigned to the control group and patients with oral-E/NR < 25% were assigned to the poor oral intake group (POI group).

### 2.4. Survey Items

Clinical data were collected from medical charts. The tumor stages were classified according to the seventh edition of the tumor-node-metastasis (TNM) classification of the Union for International Cancer Control [24]. During the postoperative hospital stay, postoperative complications included those concerning postoperative oral intake (e.g., anastomotic leakage, gastrointestinal obstruction, chyle hydrothorax, etc.), and postoperative morbidity included fever. For the anthropometry data, we defined usual body weight (UBW) as self-reported weights of patients 3–6 months preoperatively (the period from diagnosis to surgery). The previous study reported the validity of the recall method to examine the weight by 3–6 months ago [25]. We used this method in the present study. We determined the prognostic nutritional index (PNI) [3], nutritional risk index (NRI) [26], and controlling nutritional status (CONUT) [27] in patients in order to obtain an itemized list of nutrients to assess the nutritional status. PNI, NRI, and CONUT were calculated using the following formulas (Tables S1 and S2). The follow-up of patients who were surgically treated for esophageal cancer was conducted according to the protocol planned by the surgical team. In principle, they visited the hospital once in 3–6 months postoperatively and received surgical consultation, diagnostic imaging (CT or ultrasound), anthropometric test, biochemical test (e.g., nutritional status), and nutritional surveillance (by dietician). The data were recorded via medical charts. This follow-up was continued for up to five years after the surgery.

### 2.5. Statistical Analysis

Descriptive statistical analyses of variables were represented as mean ± standard deviations, number of patients, or percentages. We used multiple imputations to handle missing data because it improves accuracy and statistical power relative to other techniques to handle missing data. To impute the missing data, we constructed multiple regression models including potentially related variables and also variables correlated with outcomes. The item-level missing data rates were <25% [28] for all variables studied: (Preoperative data: Transthyretine, 19.7%; retinol-binding protein (RBP), 24.8%; NRI, 15.4%; CONUT, 0.9%; data at the 4th week postoperatively: %UBW, 1.7%; transthyretine, 0.7%; RBP, 1.7%; data at the 6th month postoperatively: C-reactive protein (CRP), 18.8%; albumin, 17.9%; PNI, 17.9%; CONUT, 23.1%; postoperative chemotherapy within one year, 7.7%; recurrence within 1 year postoperatively, 7.7%). The results across 20 imputed data sets were combined by averaging, and standard error were adjusted to reflect both within-imputation variability and between-imputation variability using Rubin’s rules [29]. The imputed data (marked by “*”) are represented as mean ± standard error. The unpaired *t*-test and the chi-square test were used to compare the data. Split-plot analysis of variance and Bonferroni adjustment for multiple comparisons were used for time-dependent data between the two study groups. Survival data were analyzed using the Kaplan–Meier survival model and calculated using the data from the surgery until the date of death or the most recent follow-up. As a sub-analysis, the two groups were analyzed for overall survival with stratification of patients by tumor stage (Stages 0–II and III–IV). The log-rank test and Bonferroni adjustment for multiple comparisons were used to determine statistical differences between the two groups. Univariate and multivariate Cox proportional hazards models were used to determine independent prognostic factors. Analytical factors included oral-E/NR at the fourth week postoperatively (≥25%, Control group vs. <25%, POI group), gender (women vs. men), tumor stages (0–II vs. III–IV), operation procedure (open esophagectomy vs. VATS), esophageal reconstruction (jejunal graft vs. gastric conduit), neoadjuvant therapy (absent vs. present), preoperative morbidities [mastication disorder (absent vs. present), dysphagia (absent vs. present), obstruction (absent vs. present)], postoperative complications (absent vs. present), postoperative morbidity (absent vs. present), postoperative meal interruption (absent vs. present), preoperative weight loss (<5% weight loss vs. ≥5% weight loss), and preoperative oral-E/total-E preoperatively (≥80% vs. <80%). We adopted preoperative weight loss for variance because we found a tendency for it to effect prognoses and nutritional status in a preceding study [30,31]. Moreover, we adopted the preoperative oral-E/total-E for variance because of the median result. For the multivariate analysis, we used the backward elimination method (elimination criteria: *p* > 0.10). SPSS version 24.0 for Windows (SPSS, Chicago, IL, USA) was used for all statistical analyses. A *p*-value < 0.05 indicated statistical significance.

## 3. Results

### 3.1. Patients’ Characteristics, Preoperative Characteristics, and Nutrient Intake between the Study Groups (POI Group vs. Control Group)

According the inclusion and exclusion criteria, 119 patients were enrolled, and two patients were excluded because their quantity of nutrients intake could not be recorded. Finally, 117 patients were analyzed in this study. The patients’ characteristics are listed in Table 1. All patients were followed up until May 2017 (mean survival time: 4.5 ± 2.3 years; survival rate, 68.4%). The patients in the POI group had significantly longer postoperative hospital stays and worse postoperative mean survival time. Additionally, the POI group exhibited a markedly higher rate of postoperative complications, morbidity, and meal interruption. However, we found no significant differences in anthropometrical data between the two groups.

### 3.2. Nutrient Intake and Nutritional Status during the Follow-Up Period between the Study Groups (POI Group vs. Control Group)

**Figure 2:**
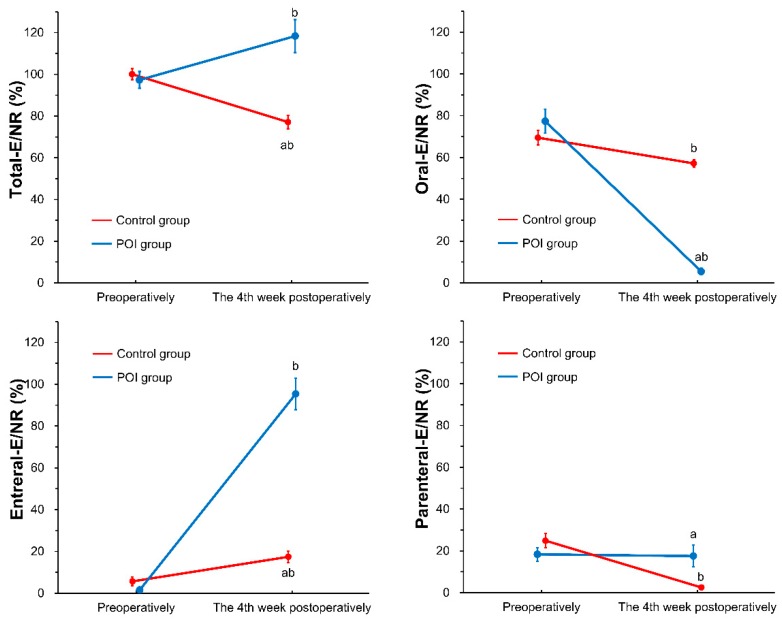
Changes of energy intake for each administration route in hospital. Differences between two study groups and two study time points were analyzed using split-plot analysis of variance and Bonferroni adjustment for multiple comparisons. Data are shown as mean ± standard error. a, *p* < 0.05 (vs. control); b, *p* < 0.05 (vs. preoperatively); POI group, poor oral intake group.

**Figure 3:**
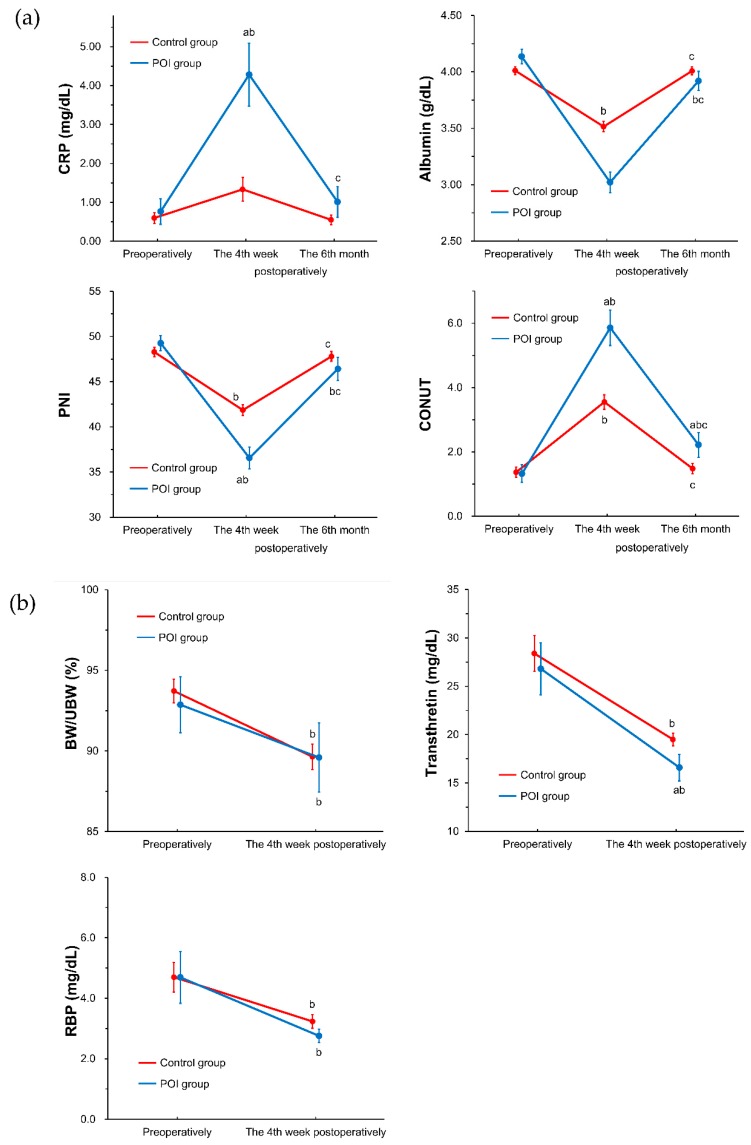
Changes of nutritional status during the follow-up period. (**a**) from preoperatively to the 6th month postoperatively, (**b**) from preoperatively to the 4th week postoperatively. Differences between two study groups and two study time points were analyzed using split-plot analysis of variance and Bonferroni adjustment for multiple comparisons. Data are shown as mean ± standard error. a, *p* < 0.05 (vs. control); b, *p* < 0.05 (vs. preoperatively); c, *p* < 0.05 (vs. the fourth week postoperatively); BW/UBW, body weight at each study time points per usual body weight.

The nutrient intakes and nutritional statuses of both study groups are shown in Table 2, Figure 2, and Figure 3a,b. The preoperative energy and protein intake did not differ significantly between the study groups [(energy) POI group, 1521 ± 319 kcal/day; control group, 1582 ± 324 kcal/day; p = 0.390 and (protein) POI group, 60.3 ± 12.8 g/day; control group, 61.4 ± 14.2 g/day; p = 0.717]. Both the groups fulfilled NRs using oral diet, EN, and PN (POI group, 97.3 ± 21.4%; control group, 100.1 ± 24.7%; p = 0.596). There were no other considerable differences in the preoperative nutrient intake for each nutritional administration route and their total. At the fourth week postoperatively, the patients in the control group principally used oral intake and displayed a shortage of energy [(energy) POI group, 1856 ± 693 kcal/day; control group, 1211 ± 413 kcal/day; *p* < 0.001 and (protein) POI group, 77.5 ± 32.4 g/day; control group, 51.3 ± 19.5 g/day; *p* < 0.001]; however, the patients in the POI group principally used EN and supplemental PN, and fulfilled NRs [(total-E/NR) POI group, 118.4 ± 41.6%; control group, 77.1 ± 30.2%; *p* < 0.001, (oral-E/NR) POI group, 5.5 ± 8.3%; control group, 57.2 ± 17.6%; *p* < 0.001, (enteral-E/NR) POI group, 95.4 ± 40.2%; control group, 17.4 ± 25.8%; *p* < 0.001, and (parenteral-E/NR) POI group, 17.5 ± 27.5%; control group, 2.5 ± 6.9%; *p* < 0.001].

The patients in the POI group showed a tendency toward higher albumin levels than those in the control group preoperatively; however, both group averages were within the normal range (Albumin*: POI group: 4.14 ± 0.06 g/dL vs. control group: 4.01 ± 0.03 g/dL, *p* = 0.086). Concerning other laboratory data and nutritional indexes, there were no significant differences between the two groups preoperatively. On the other hand, the patients in the POI group had significantly higher inflammatory indicators and worse nutritional statuses at the fourth week postoperatively. Moreover, CONUT scores continued to differ significantly at the sixth month postoperatively, mean value in POI group indicated “light malnutrition” (CONUT*: POI group: 2.22 ± 0.39 vs. control group: 1.48 ± 0.16, *p* = 0.040).

### 3.3. Long-Term Outcomes after Esophagectomy

**Figure 4:**
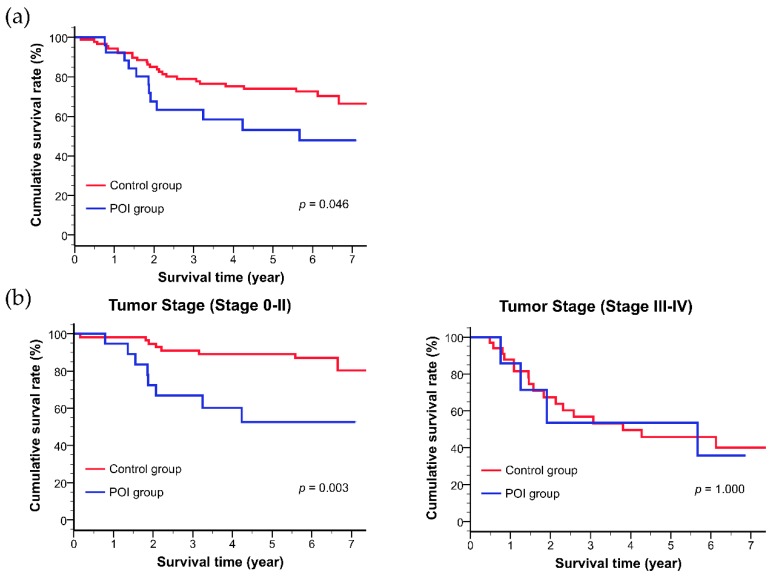
Kaplan–Meier estimates of the overall survival based on postoperative oral intake. (**a**) Overall survival, (**b**) stratification of patients by tumor stage (Stages 0–II and III–IV). Differences between the two groups were analyzed using the log-rank test and Bonferroni adjustment for multiple comparisons.

The postoperative survival rates in both study groups are shown in Figure 4a,b. The patients in the POI group had a significantly lower postoperative survival rate compared with those in the control group (mean survival time: POI group: 4.6 ± 0.5 years, control group: 6.1 ± 0.3 years, *p* = 0.046). As the result of sub-analysis, the POI group had a significantly lower postoperative survival in the patients with stage 0–II esophageal cancer (mean survival time: POI group: 4.8 ± 0.6 years, control group: 6.9 ± 0.3 years, *p* = 0.003). The results of univariate and multivariate Cox proportional hazards model are shown in Table 3. Univariate analysis showed that tumor stage (Stages III–IV), operation procedure (open esophagectomy), neoadjuvant therapy, and preoperative energy intake (<80% oral-E/total-E) had a significant negative correlation with the postoperative prognosis. Moreover, mastication disorder and energy intake at the fourth week postoperatively (POI group) showed a tendency to correlate negatively with postoperative prognosis. Based on the results of the univariate Cox proportional hazards model, we used gender, tumor stage, neoadjuvant therapy and analyzed correlation and valiance inflation factor (VIF) among variates to avoid multicollinearity. Finally, we selected mastication disorder, preoperative weight loss, and energy intake (preoperatively and at the fourth week postoperatively) as variables in the multivariate Cox proportional hazards model because these factors had a marginal significance based on the univariate Cox proportional hazards model, correlation and VIF. We performed multivariate analyses using the backward elimination method (elimination criteria: p > 0.100). Based on the multivariate Cox proportional hazards model, energy intake at the fourth week postoperatively was an independent factor negatively contributing to the postoperative prognosis [objective variables: POI group (<25% oral-E/NR), control group (≥25% oral-E/NR), adjusted hazard ratio (HR): 2.70, 95% confidence interval (CI): 1.30–5.61, *p* = 0.008].

## 4. Discussion

Our retrospective analysis of previous cases with postoperative nutritional management primarily based on the EN route suggested that the POI group demonstrated poor prognosis compared to the control group that could manage oral feeding (as a more physiological route). In this study, we revealed that assessing the oral intake at the fourth week postoperatively in patients treated surgically for esophageal cancer was not only a predictor of postoperative nutritional status but also a novel predictor of the postoperative prognosis. Hence, we believe that this study supports the validity of active nutritional management using oral intake.

In postoperative nutritional management, the most general algorithm of nutrition administration has recommended that oral intake should take priority over EN and PN [10,11]. We followed these algorithms and prioritized oral intake for perioperative nutritional management.

In nutritional management for critically ill patients such as those undergoing surgical treatment for esophageal cancer, reports about the selection of nutrition administration and nutrient quantity within at the first week postoperatively exist [32,33,34,35,36], and their results stated the importance of early EN administration and the criteria of energy administration. However, these studies referred to the exclusive use of EN and PN. Moreover, those studies contain no suggestions about a relation between oral intake postoperatively and prognosis. Currently, several studies address the topic of efficacious postoperative direct oral feeding as part of “ERAS protocol [16]”. In patients undergoing surgery for esophageal cancer, although the effect of postoperative early oral intake on long-term weight has been reported [37], its effect on postoperative prognosis is unknown. Therefore, our finding suggesting a direct relation between postoperative oral intake and prognosis of patients is important and novel.

Although patients in the POI group fulfilled NR as calculated at the fourth week postoperatively using EN by jejunostomy, they had a significantly worse nutritional status at the sixth month postoperatively and their prognosis was worse than that for control group patients. This suggests that the fulfilling of NR at the fourth week postoperatively by EN or PN was insufficient. Our study suggests that it is important for patients to attain an adequate oral intake to improve their prognosis.

Hospital stay of the patients in the POI group was longer than that of the control group because the POI group exhibited a markedly higher rate of postoperative complications, morbidity, and meal interruption. Thereby, the POI group had a large number of patients whose transition from EN or PN to oral intake was delayed. However, owing to lack of marginal significance in the results of the univariate Cox proportional hazards model for these factors, we did not use these factors in the multivariate Cox proportional hazards model. Hence, we consider that “oral-E/NR < 25%” was a valuable prognostic factor than postoperative complications because “oral-E/NR < 25%” synthetically signifies more postoperative parameters (e.g., complications and comorbidities associated with oral intake, the motivation of oral intake, and patient’s appetite).

The benefits of oral alimentation are explained in part by the action of the CPRs. The role of CPRs is to prepare the gastrointestinal tract for food digestion and absorption by promoting physiological changes before food intake [20,21]. CPRs stimulate the vagus nerve [38]; this results in the release of biological active substances (saliva [39], gastric juice [40], and exocrine pancreatic juices [41]) and hormones (insulin and glucagon [42]). Furthermore, CPRs lead to increase in stomach motility [21], changes in cardiac function [21], rise in blood pressure [21], increase in the respiratory quotient (as described in a study using rats [43]), and increase in the metabolic rate by postprandial thermogenesis [44] as a non-secretory reaction. A randomized controlled trial and an animal experiment in colorectal surgery indicated that oral and sham feedings (using chewing gum) enhance the autonomic nervous system more than enteral and parenteral routes, reducing inflammatory-based complications and length of hospital stay [45,46,47]. Additionally, in upper gastrointestinal surgery, oral feeding reduces the complications and length of stay more than enteral route [48].

Our study focused on patients surgically treated for esophageal cancer, and the study subjects were substantially different from those in the abovementioned reports. However, it is conceivable that the same benefits of the oral route of alimentation apply to our patients. In this study, we identified a novel independent predictor of the treatment outcome.

The present study suggests that a smooth transition to oral intake enhances not only the perioperative nutritional status but also the prognosis. At present, EN using jejunostomy primarily involves perioperative nutritional management in patients treated surgically for esophageal cancer to ensure adequate caloric intake. Conversely, focusing on the medical staff’s efforts on fulfilling the NRs using EN and PN could account for a delay in the transition from EN or PN to oral intake. Recently, development of the rehabilitation techniques of speech-language-hearing therapists and care food has contributed toward the improvement in patients with poor oral intake. In addition, improvement in oral intake was possible despite postoperative complications (e.g., dysphagia or obstruction and mastication disorder). Thus, a multidisciplinary team should engage even those patients who fulfill the NRs using EN and PN so that they too can re-establish an adequate oral intake during the postoperative hospital stay.

We are aware of the limitations of our study. First, the study included a relatively smaller sample size, which may confound the overall conclusion of this study. However, the 117 patients were treated at a single facility by the same surgical and nutrient management teams. Accordingly, we considered that the patients received uniform treatment and avoided problems associated with non-uniform treatment in multicenter studies. Second, our study did not investigate the differences in the expected and actual nutrient intakes to be provided in the hospital during the perioperative period and those after discharge. In a future study, we believe it will be interesting to consider a diet survey after hospital discharge and compare the oral intake between study groups at the sixth or twelfth month postoperatively.

## 5. Conclusions

In patients undergoing surgery for esophageal cancer, poor postoperative oral intake negatively affected not only their postoperative nutritional status but also their overall prognosis. It is necessary to improve the adequacy of oral intake postoperatively for patients with esophageal cancer. Hence, we believe that this study supports the validity of efficacious postoperative direct oral feeding as part of the “ERAS protocol”.

## Figures and Tables

**Table 1:** Patients’ characteristics.

	Total (*n* = 117)	Control Group (*n* = 89)	POI Group (*n* = 28)	*p*-Value (Control vs. POI)
Gender (Men/Women)	88/29	68/21	20/8	0.595
Age (years)	64.0 ± 7.9	63.7 ± 7.5	65.0 ± 9.2	0.435
Height (cm)	163.4 ± 7.8	164.0 ± 7.5	161.5 ± 8.6	0.137
Weight (kg)	56.0 ± 8.9	56.0 ± 9.4	56.0 ± 7.5	1.000
BMI (kg/m^2^)	20.9 ± 2.7	20.8 ± 2.7	21.5 ± 2.7	0.203
UBW (kg)	60.2 ± 10.0	59.9 ± 9.5	61.1 ± 11.3	0.567
Preoperative morbidities				
Mastication disorder	10 (8.5%)	7 (7.9%)	3 (10.7%)	0.638
Dysphagia	10 (8.5%)	9 (10.1%)	1 (3.6%)	0.280
Obstruction	42 (35.9%)	33 (37.1%)	9 (32.1%)	0.635
Glucose intolerance	30 (20.4)	23 (25.8%)	7 (25.0%)	0.929
Hypertension	32 (27.4%)	24 (27.0%)	8 (28.6%)	0.868
Dyslipidemia	8 (6.8%)	5 (5.6%)	3 (10.7%)	0.351
Tumor stage (0/IA/IB/IIA/IIB/IIIA/IIIB/IV)	13/24/9/12/17/24/0/18	9/17/6/11/13/21/0/12	4/7/3/1/4/3/0/6	0.502
Operative procedure (VATS/Open esophagectomy)	76/41	56/33	20/8	0.411
Esophageal reconstruction (Gastric conduit/Jejunal graft)	110/7	84/5	26/2	0.767
Postoperative complications	77 (65.8%)	51 (57.3%)	26 (92.9%)	<0.001
Postoperative morbidity	61 (52.1%)	39 (43.8%)	22 (78.6%)	0.001
Postoperative meal start (POD)	13.9 ± 13.6	10.1 ± 3.9	26.1 ± 23.7	0.002
Postoperative meal interruption	61 (52.1%)	39 (43.8%)	22 (78.6%)	0.001
Neoadjuvant therapy	60 (51.3%)	48 (53.9%)	12 (42.9%)	0.306
Postoperative hospital stay (days)	36.3 ± 26.3	26.7 ± 8.3	66.9 ± 38.4	<0.001
Postoperative chemotherapy within 1 year postoperatively *	55 (47.0%)	43 (48.3%)	11 (39.3%)	0.403
Recurrence within 1 year postoperatively *	37 (31.6%)	26 (29.2%)	10 (35.7%)	0.516
Postoperative mean survival time (years)	4.5 ± 2.3	4.7 ± 2.2	3.5 ± 2.4	0.016

Data are expressed as the mean ± standard deviation or as the number of patients. Imputed data (*) are expressed as the mean ± standard error or as number of patients. Differences among two groups were analyzed using the unpaired *t*-test and chi-square test. BMI, body mass index; UBW, usual body weight; VATS, video-assisted thoracoscopic esophagectomy.

**Table 2:** Comparison of nutrient intake in the hospital between the study groups.

	Total (*n* = 117)	Control Group (*n* = 89)	POI Group (*n* = 28)	*p*-Value (Control vs. POI)
**Preoperatively**				
Energy intake in the hospital (kcal/day)	1567 ± 323	1582 ± 324	1521 ± 319	0.390
Via oral route (kcal/day)	1136 ± 505	1115 ± 520	1204 ± 458	0.422
Via enteral route (kcal/day)	70 ± 262	86 ± 295	21 ± 81	0.065
Via parenteral route (kcal/day)	361 ± 429	381 ± 462	297 ± 302	0.369
Protein intake in the hospital (g/day)	61.1 ± 13.8	61.4 ± 14.2	60.3 ± 12.8	0.717
Per preoperative weight (g/kg)	1.12 ± 0.31	1.12 ± 0.33	1.09 ± 0.26	0.656
Via oral route (g/day)	46.1 ± 21.0	45.2 ± 21.9	48.9 ± 18.1	0.415
Via enteral route (g/day)	3.0 ± 11.6	3.7 ± 13.2	0.8 ± 2.9	0.056
Via parenteral route (g/day)	12.1 ± 13.8	12.5 ± 14.6	10.6 ± 10.9	0.524
**The 4th week postoperatively**				
Nutrient requirement (kcal/day)	1599 ± 192	1605 ± 200	1578 ± 192	0.514
Energy intake in the hospital (kcal/day)	1366 ± 564	1211 ± 413	1856 ± 693	<0.001
Via oral route (kcal/day)	716 ± 434	913 ± 279	88 ± 156	<0.001
Via enteral route (kcal/day)	557 ± 698	260 ± 373	1501 ± 654	<0.001
Via parenteral route (kcal/day)	92 ± 240	38 ± 103	268 ± 414	<0.001
Protein intake in the hospital (g/day)	57.6 ± 26.7	51.3 ± 19.5	77.5 ± 32.4	<0.001
Per postoperative weight (g/kg)	1.10 ± 0.50	0.99 ± 0.43	1.45 ± 0.57	<0.001
Via oral route (g/day)	30.8 ± 20.6	39.4 ± 15.4	3.5 ± 5.8	<0.001
Via enteral route (g/day)	24.4 ± 31.0	11.4 ± 16.8	65.6 ± 30.1	<0.001
Via parenteral route (g/day)	2.7 ± 8.1	0.9 ± 3.5	8.4 ± 14.1	0.010

Data are expressed as the mean ± standard deviation. Differences among two groups were analyzed using the unpaired *t*-test. POI group, poor oral intake group.

**Table 3:** Univariate and multivariate Cox regression analyses of prognostic factors in patients with esophageal cancer.

Factors	Objective Variables	Control	Univariate Analysis		Multivariate Analysis	
			Crude HR (95% CI)	*p*-Value	Adjusted HR (95% CI)	*p*-Value
Gender	Male	Female	1.85 (0.77–4.45)	0.167		
Age	≥65	<65	1.22 (0.64–2.32)	0.548		
Tumor stage	III–IV	0–II	3.51 (1.82–6.74)	<0.001	3.72 (1.92–7.20)	<0.001
Operation procedure	Open esophagectomy	VATS	2.23 (1.17–4.26)	0.015		
Esophageal reconstruction	Jejunal graft	Gastric conduit	0.91 (0.22–3.77)	0.905		
Neoadjuvant therapy	Present	Absent	1.96 (1.01–3.82)	0.048	2.33 (1.16–4.70)	0.018
Preoperative morbidities						
Mastication disorder	Present	Absent	2.25 (0.87–5.78)	0.093		
Dysphagia	Present	Absent	1.83 (0.65–5.18)	0.253		
Obstruction	Present	Absent	1.42 (0.74–2.75)	0.292		
Postoperative complications	Present (77)	Absent (40)	0.80 (0.41–1.54)	0.498		
Postoperative morbidity	Present (61)	Absent (56)	1.32 (0.69–2.53)	0.410		
Postoperative meal interruption	Present (61)	Absent (56)	1.07 (0.56–2.03)	0.847		
Preoperative weight loss	≥5% weight loss	<5% weight loss	1.70 (0.88–3.28)	0.112		
Energy intake at preoperatively	<80% oral-E/total-E	≥80% oral-E/total-E	2.14 (1.12–4.07)	0.021		
Energy intake at the 4th week postoperatively	<25% oral-E/NR (POI group)	≥25% oral-E/NR (Control group)	1.99 (1.00–3.98)	0.051	2.70 (1.30–5.61)	0.008

The univariate Cox proportional hazards model included gender, age, tumor stage, operation procedure, neoadjuvant therapy, preoperative morbidities (mastication disorder, dysphagia and obstruction), postoperative complications, postoperative morbidity, postoperative meal interruption, preoperative weight loss, and energy intake (at preoperatively and the fourth week postoperatively). The multivariate Cox proportional hazards model [backward elimination method (elimination criteria: *p* > 0.10)] included gender, tumor stage, operation procedure, neoadjuvant therapy, mastication disorder, preoperative weight loss, and energy intake (at preoperatively and the fourth week postoperatively).

## Supplementary Materials

The following are available online at https://www.mdpi.com/2072-6643/11/6/1338/s1, Table S1: Equations of BEE, PNI, and NRI, Table S2: Equations of CONUT.
