# Supplementary material for: Effect of Postoperative Oral Intake on Prognosis for Esophageal Cancer
Source: Nutrients. 2019 Jun 14;11(6):1338. doi: 10.3390/nu11061338 (PMC6627190; doi:10.3390/nu11061338)
Supplement: Supplementary file 1 [file nutrients-11-01338-s001.pdf]

Supplement data

Table S1: Equations of BEE, PNI, and NRI

1. Harris–Benedict equation [Basal energy expenditure (BEE)]

Men: $BEE = 66.47 \times 13.75 \times Weight\ (kg) + Height\ (cm) - 6.76 \times Age\ (years)$

Women: $BEE = 655.1 + 9.65 \times Weight\ (kg) + 1.85 \times Height\ (cm) - 4.68 \times Age\ (years)$

2. Prognostic nutritional index (PNI)

$$PNI = 10 \times Albumin\ (g/dL) + 0.005 \times TLC\ (cells/mm^3)$$

3. Nutritional risk index (NRI)

$$NRI = 10.7 \times Albumin\ (g/dL) + 0.00039 \times TLC(cells/mm^3) + 0.11 \times Zinc\ (\mu g/dL) - 0.44 \times Age(years)$$

Table S2: Equations of CONUT

$$CONUT\ score = Albumin\ score + TLC\ score + T{-}\ Cho\ score$$

	Under nutrition degree			
Parameter	Normal	Light	Moderate	Severe
CONUT score	0–1	2–4	5–8	9–12
Albumin (g/dL)	3.5–4.5	3.0–3.49	2.5–2.9	< 2.5
Albumin score	0	2	4	6
TLC (cells/mm^3)	> 1,600	1,200–1,599	800–1,199	< 800
TLC score	0	1	2	3
T–Cho (mg/dL)	>180	140–180	100–139	< 100
T–Cho score	0	1	2	3
